# ADVANCED MEGAESOPHAGUS TREATMENT: WHICH TECHNIQUE OFFERS THE BEST RESULTS? A SYSTEMATIC REVIEW

**DOI:** 10.1590/0102-6720202400016e1809

**Published:** 2024-07-01

**Authors:** Paulo Sérgio CHAIB, Gloria de Almeida TEDRUS, José Luís Braga de AQUINO, José Alexandre MENDONÇA

**Affiliations:** 1 Pontifícia Universidade Católica de Campinas, Postgraduate Program of Health Sciences, Campinas (SP), Brazil.

**Keywords:** Esophageal Achalasia, Digestive System Surgical Procedures, Esophagectomy, Myotomy, Treatment Outcome, Systematic Review, Acalásia Esofágica, Procedimentos Cirúrgicos do Sistema Digestório, Esofagectomia, Miotomia, Resultado do Tratamento, Revisão Sistemática

## Abstract

**BACKGROUND:**

Advanced megaesophagus predisposes to risks of malnutrition infections and cancer, in addition to having a significant impact on quality of life. There is currently no consensus in the literature regarding the best surgical option for advanced megaesophagus, although there is a predilection for esophagectomy, despite this surgery being associated with significant morbidity and mortality. Other surgical procedures, such as esophageal mucosectomy and Heller cardiomyotomy, have been proposed with good results.

**AIMS:**

To conduct a systematic review and meta-analysis of the literature on the surgical treatment of advanced megaesophagus.

**METHODS:**

Databases used included PubMed, Latin American and Caribbean Health Sciences Literature (Lilacs), Embase and Medical Literature Analysis and Retrieval System Online (MedLine), as well as reference research. Two reviewers selected the articles independently.

**RESULTS:**

A total of 14 articles were chosen, which included 1,862 patients. The studies were divided into two groups: laparoscopic cardiomyotomy with fundoplication (213 patients) and major surgeries (1,649 patients). The studies yielded mostly good or excellent results regarding late outcomes in both groups. However, there was significant morbidity associated with the major surgeries group.

**CONCLUSIONS:**

Laparoscopic Heller myotomy can be performed on patients with advanced megaesophagus, with lower rates of complications and mortality compared to major surgeries, with reservations regarding late outcomes results.

## RESUMO

**RACIONAL:**

O megaesôfago avançado predispõe riscos clínicos de desnutrição, infecções e neoplasias, além de impacto significativo na qualidade de vida. Não há um consenso atual na literatura ante a melhor opção de seu tratamento cirúrgico, embora haja predileção pela esofagectomia, cirurgia de significativa morbimortalidade associada. Outras modalidades cirúrgicas têm sido propostas, com bons resultados, como a mucosectomia esofágica e a cardiomiotomia laparoscópica à Heller.

**OBJETIVOS:**

Realizar uma revisão sistemática com metanálise da literatura acerca do tratamento cirúrgico do megaesôfago avançado.

**MÉTODOS:**

As bases de dados utilizadas foram PubMed, Lilacs, Embase e MedLine, além de pesquisas de referências relacionadas. Os artigos foram selecionados por dois revisores independentemente.

**RESULTADOS:**

Foram selecionados 14 artigos, que incluem 1.862 pacientes. Os estudos foram divididos em dois grupos: cardiomiotomia laparoscópica com fundoplicatura (213 pacientes) e cirurgias de grande porte (1.649 pacientes). Os estudos analisados evidenciam que ambos os grupos apresentaram resultados semelhantes quanto ao desfecho tardio, considerado majoritariamente bom ou excelente, no entanto, houve significativa morbimortalidade associada ao grupo de cirurgias maiores.

**CONCLUSÕES:**

A cardiomiotomia laparoscópica com fundoplicatura pode ser realizada no megaesôfago avançado, com taxas de complicações e mortalidade reduzidas frente às cirurgias de grande porte, porém, com ressalvas quanto ao desfecho tardio a longo prazo.

## INTRODUCTION

Achalasia is an inflammatory neurodegenerative disorder of the esophagus, which, through the destruction of neurons in the myenteric plexus of the distal esophagus, prevents relaxation of the lower esophageal sphincter (LES) and incoordination of esophageal peristalsis^24,35,38^. It is defined as a denervation esophagopathy with broad-spectrum dysmotility^13^ hampering emptying and dilation of the esophagus, clinically characterized as megaesophagus^16^.

Terminal achalasia occurs in around 10–15% of all patients with the disease^22^ and is characterized by advanced megaesophagus (grades III and IV — Resende/Mascarenhas classification), with dolichomegaesophagus (“sigmoid-esophagus”), significant tortuosity, esophageal diameter above 6 cm. It occurs due to failure of previous treatments^9,24^. These patients present conditions with severe symptoms, which directly impact their quality of life. Furthermore, they commonly present life-threatening complications, such as malnutrition, immunodeficiency, repetitive bronchoaspiration and a high risk of developing sepsis and neoplasms^9,24,38^.

In the world literature, there is still no consensus on the best surgical option for definitive treatment of advanced megaesophagus. Subtotal esophagectomy is still suggested as the main treatment option for advanced megaesophagus in elective cases; however, this procedure presents significant morbidity (19 to 69%) and mortality rates (0 to 9%)^1,23,34^.

Alternative techniques such as esophageal mucosectomy, developed by Aquino et al.^3^, present significantly better results when compared to esophagectomy in the treatment of terminal achalasia. On the other hand, it involves carrying out a major abdominal surgery with all the risks inherent to such procedure^5,6^.

With the purpose of achieving a less morbid treatment for these patients, who may already be weakened by this disease, some authors propose performing laparoscopic Heller cardiomyotomy, with results generally considered satisfactory. However, the accumulated risk of long-term neoplasia, regurgitation and bronchoaspiration is questioned when keeping the esophagus *in situ*, an inert pouch, and impaired emptying^12,21^.

The present study is justified based on the need to provide a better understanding of the different types of surgical treatments for advanced megaesophagus, considering the risks and postoperative morbidity and mortality, as well as results, effectiveness, and late outcomes. A more incisive guide to allow the surgeon’s selection of the best surgical treatment for advanced megaesophagus is required.

Our work aimed to carry out a systematic review with meta-analysis on the surgical treatment of advanced megaesophagus, with view at describing the main modalities currently in use and whose scope involves the comparative assessment of such modalities’ morbidity, mortality, complications rates and outcomes and late results.

## METHODS

Our systematic review was conducted in accordance with the recommendations and following the Preferred Reporting Items for Systematic Reviews and Meta-Analyses (PRISMA)^25^ method checklist. After a prepared question, the Patient or Problem, Intervention, Control or Comparison, Outcomes (PICO) strategy was used in order to identify the outcome of advanced megaesophagus surgical treatment.

The eligibility criteria included:

Participant type (P): patients diagnosed with advanced megaesophagus.

Types of intervention (I and C): esophagectomy, esophageal mucosectomy, Serra-Doria surgery, Heller cardiomyotomy. The types of intervention were not applicable to control patients. The survey included a review of non-comparative studies.

Types of outcomes (O): surgical outcomes considering morbidity, mortality, complications, length of stay, late results, effectiveness, quality of life.

The aim of our work was to search for the most current forms of surgical treatment for advanced megaesophagus, and, hence it was decided to include only articles published in the last ten years. Furthermore, as this is an uncommon disease, the included articles had to have a sample of patients greater than or equal to eight cases, submitted to previous treatments or not.

### Inclusion criteria

- Studies that included patients with advanced achalasia and/or advanced megaesophagus of any etiology (grades III and IV, sigmoid esophagus, terminal achalasia), undergoing any type of definitive surgical treatment.
- Studies with patients aged ≥18 years.
- Studies with a patient sample greater than or equal to eight cases.
- Cohort studies, cross-sectional studies, case series, randomized or non-randomized clinical trials.
- Studies evaluated and selected by two independent reviewers.
- Studies written in English, Portuguese or Spanish.
- Articles published from 2012 onwards.

### Exclusion criteria

- Studies with patients without a diagnosis of advanced achalasia/advanced megaesophagus.
- Studies with patients diagnosed with advanced achalasia/megaesophagus undergoing definitive non-surgical treatments.
- Case reports, correspondence, animal models, literature reviews, systematic reviews or meta-analyses.
- Studies without full text.

### Selection of articles

A search using a predefined strategy was carried out in electronic databases by two reviewers independently. Any disagreement between reviewers was settled by consensus after discussion with a third researcher. The articles were screened according to previously established inclusion/exclusion criteria. When similar articles from the same institution were found, the article with a larger patients’ sample was selected.

Two separate reviews were carried out, one qualitative and one quantitative (meta-analysis). The latter compared the following outcomes: morbidity/complications, mortality and late outcomes considered good or excellent.

### Database

The databases searched electronically were PubMed, Medical Literature Analysis and Retrieval System Online (MedLine), Latin American and Caribbean Health Sciences Literature (Lilacs) and Embase. The structured search strategy involved the following terms: (esophageal achalasia) OR (achalasia) OR (end-stage achalasia) OR (megaesophagus) OR (advanced megaesophagus) OR (sigmoid-esophagus) AND (surgery) OR (minimally invasive surgery) OR (laparoscopic myotomy) OR (laparoscopic heller myotomy) OR (laparoscopic cardiomyotomy) OR (serra-doria surgery) OR (esophagectomy) OR (esophageal resection) OR (mucosectomy) OR (esophageal mucosectomy) AND (groups) OR (trial) OR (surgery) OR (randomly) OR (randomized) OR (clinical trial) OR (comparative study) OR (controlled clinical trial) OR (randomized controlled trial) AND (surgery outcomes) OR (outcomes) OR (morbidity) OR (mortality) OR (follow-up) OR (quality of life).

The search for references of relevant articles and abstracts published in conference proceedings was also considered in the review. The last survey was carried out in June 2022. The survey results are reported in Table 1.

**Table 1:** Search results.

Data base	Articles found	Selected articles
	n	n
PubMed	127	2
MEDLINE	260	2
Lilacs	247	3
Others	11	6
Total	969	14

### Bias risk analysis methodology in non-randomized studies

Non-randomized studies were subjected to the risk of bias analysis using the ROBINS-I platform (Risk Of Bias In Non-randomized Studies — of Interventions); the same methodology was used to assess the risk of bias of a randomized study^32^.

### Statistical analysis

Statistical analysis was carried out through the development of a meta-analysis using the Cochrane Review Manager (RevMan) software (https://training.cochrane.org/online-learning/core-software/revman), organized in forest plot and funnel plot graphs. Statistical significance was considered at p<0.05 and confidence interval at 95% (95%CI)^15^. The heterogeneity of the studies was assessed using the I test².

## RESULTS

The total number of articles assessed was 969 and the total number of articles selected for the work which met the pre-established inclusion/exclusion criteria was 14, totaling 1,862 patients.

The database screening involved 958 articles. Out of these, after excluding duplicate articles and those that were not relevant to the work, 84 articles were selected for full text reading. Finally, eight articles were selected for the work. The remaining articles were excluded because they did not present a scope relevant to the work or because data were missing in connection with the objective of this study.

Within the data search carried out, some abstracts published in conference proceedings were identified and reviewed. A reference search for relevant articles was also carried out. A total of 11 pertinent articles were found and, after application of the exclusion criteria, six articles were finally selected.

The papers were separated into two large groups: patients undergoing cardiomyotomy (six articles; n=213) and patients undergoing major surgeries (nine articles; n=1,649), and this group included the following surgeries: esophagectomy, subtotal esophagectomy, transhiatal esophagectomy, minimally invasive esophagectomy, esophageal mucosectomy, Serra-Doria esophagocardioplasty. The major surgeries mentioned above were considered as such because they necessarily involved a digestive anastomosis.

The article by Tassi et al.^33^ was allocated to both groups as it encompasses patients studied using both surgical modalities. Some studies within the group of major surgeries presented results involving more than one surgical technique^2,11,20^.

Of the 14 studies selected for the work, one^18^ was not eligible for meta-analysis due to missing data. Hence, the meta-analysis included a total of 13 articles and 686 patients.

### Preferred Reporting Items for Systematic Reviews and Meta-Analyses (PRISMA) flowchart

**Figure 1:**
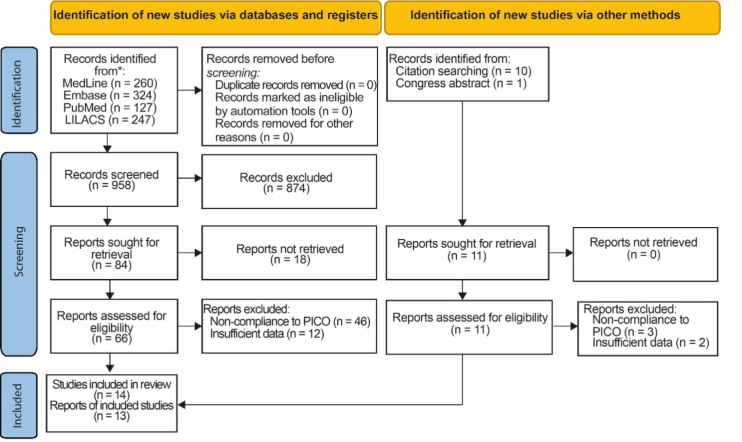
Preferred Reporting Items for Systematic Reviews and Meta-Analyses (PRISMA) flowchart^25^.

The selection and inclusion of articles is shown in the PRISMA flowchart (Figure 1).

### Qualitative results: systematic review

The results were summarized in tables, as explained below. The surgeries were divided into two large groups, named “cardiomyotomy” and “major surgeries” (Tables 2A, 2B, 3A, 3B). The objective items of study and comparison in this work were the following: study design, type of surgical treatment performed, number of patients, average age, gender, definition and classification of advanced achalasia/megaesophagus, general complications and morbidity, mortality, time of hospitalization, average follow-up time and late results.

**Table 2A:** Systematic review of studies of cardiomyotomy with fundoplication for advanced megaesophagus.

Study	Study design	Treatment carried out	No. of patients	Average age (years)	Gender (M/F)	Classification of achalasia
Panchanatheeswaran et al.^26^	Retrospective cohort	Laparoscopic Heller cardiomyotomy + antireflux procedure	8	39.5	M50% F50%	“Sigmoid esophagus”
Pantanali et al.^27^	Retrospective cohort	Laparoscopic Heller cardiomyotomy + Pain fundoplication	11	56	M6 F5	>10 cm (diameter)
Simić et al.^31^	Retrospective cohort	Laparoscopic Heller-Dor cardiomyotomy	10	51	-	“Sigmoid esophagus”
Rosemurgy et al.^29^	Retrospective cohort	Laparoscopic Heller cardiomyotomy + anterior fundoplication	10 III: 3 IV: 7	III: 61 IV 56	III: M0M3 IV: F4M3	III: >6 cm, IV: >3 esophageal curves and >6 cm (diameter)
Costantini et al.^7^	Retrospective cohort	Laparoscopic Heller-Dor cardiomyotomy	142 III: 87 IV: 55	46	-	grade III: >6cm (diameter) grade IV: “sigmoid-shaped esophagus”
Tassi et al.^33^	Retrospective cohort	Laparoscopic Heller-Dor “Pull-down” cardiomyotomy (CLH) x Esophagectomy (E)	CLH: 32	CLH: 57	CLH: M34.37% F65.62%	“End-stage achalasia”

M: male; F: female.

**Table 2B:** Systematic review of cardiomyotomy studies with fundoplication for advanced megaesophagus.

Study	Complications/morbidity	Mortality	Length of stay (days)	Average follow-up time	Late results
Panchanatheeswaran et al.^26^	Morbidity 0% 1 iatrogenic intraoperative complication	None	4.25	19.5 months	100% Excellent or Good (50–50%)
Pantanali et al. ^27^	Morbidity 0%	None	1	31.5 months	72.8% Excellent or Good
Simić et al.^31^	Morbidity 0% 1 mucosal perforation 1 trocar bleeding 1 wound infection	None	2	28 months	94.4% resolution of dysphagia
Rosemurgy et al.^29^	Intraoperative: 0 Postoperative period: 1 (atelectasia)	None	III: 4 IV: 3	27 months	III: 33% Excellent 66% Good IV: 25% Excellent 75% Good
Costantini et al.^7^	Morbidity 4.7% 22 mucosal perforations 1 splenic injury 2 Trocar bleeding	0.1% (AMI)	-	62 months	89.5% Good outcome III: 90.8% IV 76.4% Failure: III 9.2% IV 23.6%
Tassi et al.^33^	CLH: 12.5% 1 mucous fistula 1 mucous membrane 1 hyper-dysphagia 1 hyper competent fundoplication	There were none in both groups	CLH: 6	CLH: 68 months	CLH: 46.87% Excellent 34.37% Good

CLH: Laparoscopic Heller-Dor “Pull-down” cardiomyotomy; AMI: acute myocardial infarction.

**Table 3A:** Systematic review of major surgery studies for advanced megaesophagus.

Study	Study design	Treatment carried out	No. of patients	Average age (years)	Gender (M/F)	Classification of achalasia
Molena et al.^18^	Retrospective cohort	Esophagectomy	963	54.6	M49.01% F50.99%	-
Felix et al*.* ^10^	Case series	Transhiatal esophagectomy	11	44	M8 F3	“*sink trap megaesophagus*”
Oliveira et al.^20^	Retrospective cohort	Transhiatal esophagectomy (THE) x Mucosectomy (ME)	40 THE: 23 ME: 17	-	-	Advanced megaesophagus
Aquino et al.^4^	Retrospective cohort	Serra-Doria esophagocardioplasty	19	63 a 78	M14 F5	Grades III and IV (Rezende Classification)
Aquino et al.^2^	Retrospective cohort	Esophageal mucosectomy (ME) x Transhiatal esophagectomy (THE)	229 ME: 115 THE: 114	15-76 years	M70.3% F29.7%	Advanced megaesophagus
Crema et al.^8^	Cohort	Transhiatal VLP esophagectomy with vagus nerve preservation	136	59.3	M59.5% F40.45%	Advanced megaesophagus
Fontan et al.^11^	Randomized clinical trial	Open transhiatal esophagectomy vs. VLP	30 open: 15 VLP: 15	open: 47.2 VLP: 44.1	open: M8 F7 VLP: M11 F14	Grades III and IV (Rezende Classification)
Torres-Landa et al.^34^	Retrospective cohort	Esophagectomy (E)	209	56	M51.8% F48.2%	-
Tassi et al.^33^	Retrospective cohort	Laparoscopic Heller-Dor “Pull-down” cardiomyotomy (CLH) x Esophagectomy (E)	E: 12	E: 59	E: M62.5% F37.5%	“End-stage achalasia”

M: male; F: female; VLP: videolaparoscopic.

**Table 3B:** Systematic review of studies of major surgeries for advanced megaesophagus.

Study	Complications/morbidity	Mortality	Length of stay (days)	Average follow-up time	Late results
Torres-Landa et al.^34^	Morbidity 43.5% Readmission 2.2% Reoperation 6.7% Sepsis 9.5% Pneumonia 12.4% Blood transfusion 20.5%	None	10	1 month	Not assessed
Tassi et al.^33^	E: 43.75% 3 anastomosis fistulas 1 pyloroplasty fistula 1 pleural empyema 1 acute respiratory failure	There were none in both groups	E: 23	E: 61 months	E: 37.5% excellent 25% good

### Quantitative results: meta-analysis

**Figure 2:**
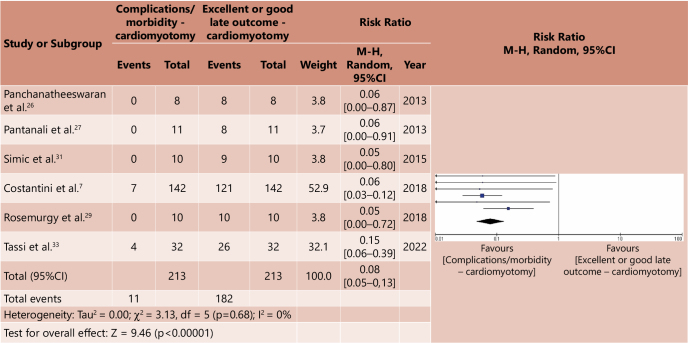
Comparative meta-analysis between morbidity/complications x good or excellent late outcome in cardiomyotomy – Forest plot^15^.

**Figure 3:**
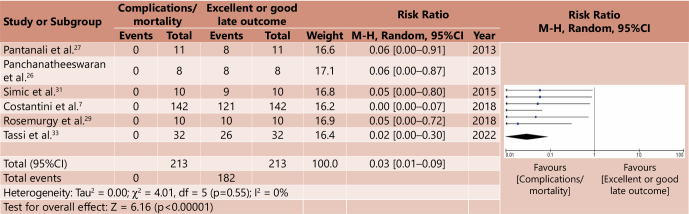
Comparative meta-analysis between mortality x good or excellent late outcome in cardiomyotomy — Forest plot^15^.

**Figure 4:**
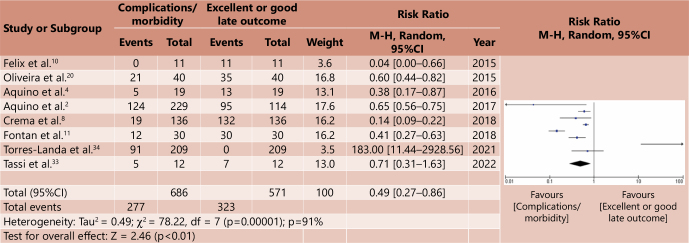
Comparative meta-analysis between morbidity/complications x good or excellent late outcome in major surgeries – Forest plot^15^.

**Figure 5:**
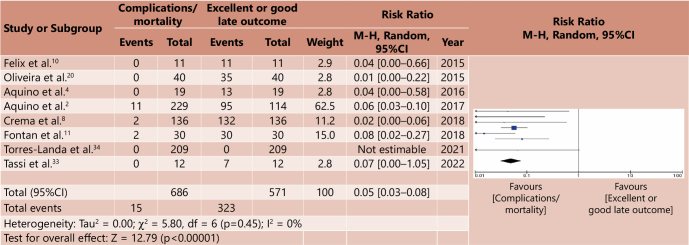
Comparative meta-analysis between mortality x good or excellent late outcome in major surgeries – Forest plot^15^.

The meta-analysis was carried out based on a systematic correlation between morbidity/complications and mortality and late outcomes considered good or excellent, for both groups. In this way, four forest plot graphs were generated, two for the cardiomyotomy group and two for the major surgery group (Figures 2, 3, 4, 5). Analysis of the risk of bias of the selected studies was carried out based on the ROBINS-I platform, as shown in Table 4. A correlation was made between the relative risk (RR) generated from the meta-analyses for the outcomes assessed. Table 5 demonstrates such comparative analysis.

**Table 4:** Risk of bias in the studies included in the meta-analysis^32^.

Study	Risk of bias domains							
	D1	D2	D3	D4	D5	D6	D7	Overall
Rosemurgy et al.^29^	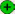	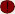	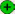	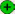	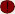	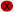	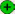	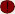
Fontan et al.^11^	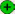	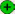	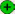	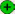	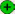	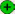	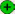	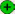
Pantanali et al.^27^	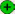	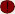	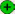	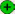	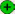	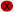	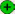	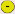
Torres-Landa et al.^34^	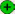	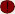	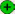	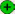	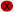	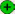	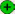	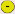
Aquino et al.^4^	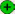	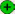	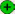	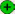	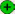	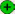	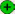	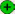
Oliveira et al.^20^	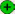	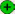	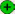	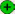	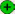	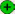	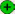	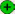
Crema et al.^8^	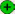	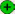	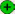	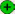	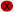	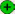	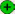	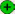
Aquino et al.^2^	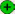	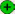	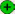	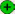	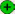	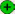	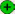	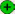
Felix et al.^10^	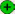	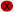	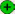	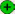	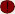	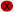	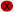	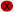
Panchanatheeswaran et al.^26^	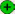	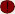	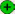	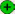	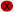	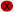		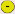
Simic et al.^31^	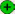	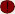	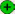	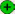	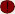	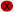		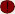
Molena et al.^18^	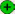	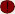	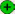	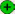	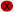	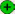		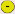
Costantini et al.^7^	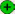	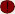	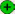	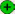	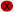	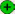		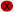
Tassi et al.^33^	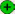	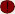	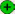	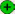	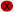	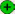		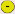
	Domains:						Judgment	
	D1: Bias due to confounding.						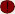	Critical
	D2: Bias due to selection of participants.							
	D3: Bias in classification of interventions.						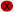	Serious
	D4: Bias due to deviations from intended interventions.							
	D5: Bias due to missing data.						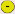	Moderate
	D6: Bias in measurement of outcomes.							
	D7: Bias in selection of the reported result.						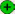	Low

**Table 5:** Relative risk between the cardiomyotomy and major surgery groups compared to the comparative analysis of morbidity/complications and mortality x good or excellent late outcomes.

Groups	Morbidity/complications	Mortality
Cardiomyotomy	0.08	0.03
Major surgeries	0.49	0.05

## DISCUSSION

From the review of the data gathered in this work, we can detect some significant aspects of the surgical treatment of advanced megaesophagus. The systematic review and meta-analysis carried out allow for sufficient data to be provided for an in-depth analysis of the two large treatment groups evaluated.

The late results of the cardiomyotomy group were considered satisfactory by the authors (good or excellent) and are indeed very impressive. Although most studies involved less than 12 patients, even in studies with a greater number of patients, such as those by Costantini et al.^7^ (142 patients), and Tassi et al.^33^ (32 patients), these numbers reached rates of 89.5 and 81.24% respectively, in a late assessment with more than 60 months follow-up.

Complications and morbidity in the major surgery group were significantly higher than in the cardiomyotomy group. In most studies, between 40 and 50% of patients underwent this form of treatment; in one series it reached 69.2%.

Late results in this group, unlike the case of the cardiomyotomy group, were assessed heterogeneously. In general, they were also considered mostly satisfactory in all the series.

From the comparative meta-analysis between complications/morbidity and good or excellent late outcomes in the cardiomyotomy group, it was concluded that there is a low impact of morbidity/complications in relation to cardiomyotomy with fundoplication for patients with advanced megaesophagus. The RR was 0.08 (p<0.00001, 95%CI 0.05–0.13).

In the comparative analysis between mortality and good or excellent late outcomes in the cardiomyotomy group, the RR for this outcome was 0.03 (p<0.00001, 95%CI 0.01–0.09), that is, there is also a considerably low impact of the outcome in this analysis.

When evaluating the comparative review between morbidity/complications and good or excellent late outcomes in the group of major surgeries, there is a relatively low impact of morbidity/complications compared to good or excellent late outcomes for major surgeries, with a RR of 0.49 (p=0.01, 95%CI 0.27–0.86).

The comparative analysis between mortality and good or excellent late outcomes in the group of major surgeries also shows that there is a low impact of mortality compared to the late outcome, with a RR of 0.05 (p<0.00001, 95%CI 0.03–0.08).

When comparing these two groups, it can be concluded that they both present similar results from their treatments, with a low impact on morbidity and mortality and a tendency to favorable late outcomes. The RR of complications in relation to a favorable late outcome in the cardiomyotomy group was 0.08 and that of mortality was 0.03. This risk was considerably lower than the RR of complications and mortality in relation to the favorable late outcome in the major surgery group, 0.49 and 0.05, respectively.

This allows us to conclude that both modalities show good general surgical results; however, patients undergoing cardiomyotomy have lower risks of developing complications and/or mortality, compared to patients undergoing major surgeries, as already assessed in the systematic review of this study. Furthermore, there are other considerable underlying factors in this framework, such as shorter hospital stays, reduced hospital costs and lower demand for treatment complexity — when compared to major surgeries.

An important caveat must be made regarding the term “definitive treatment”, since most studies present a short to medium-term follow-up period. There are still questions regarding relapses and/or progression of the disease in this treatment modality.

The data found are in accordance with the world literature. Meta-analysis by Niño-Ramírez et al.^19^ involving 5,492 patients undergoing laparoscopic Heller cardiomyotomy revealed a 4.9% rate of adverse events, most of them associated with perforation of the esophageal mucosa. The 30-day mortality rate in this group of patients was 0.09%.

The systematic review with meta-analysis by Orlandini et al.^22^ evaluated 350 patients who underwent surgical Heller cardiomyotomy for advanced sigmoid megaesophagus with the following late results rates: complication, 8.0%; mortality, 0.8%; retreatment requirement, 12.8%; and 76.2% probability of results considered good or excellent after this surgical procedure. It was concluded that this surgical modality is acceptable as definitive treatment for patients with advanced/sigmoid megaesophagus, because it avoids esophagectomy, has low morbidity and mortality rates and low rates of retreatment requirements^22^.

In a similar review, Herbella and Patti^14^, in an assessment of 122 patients in eight studies, found an average of 79% good or excellent late results without any associated mortality, in patients with advanced megaesophagus undergoing Heller cardiomyotomy. They concluded that laparoscopic Heller cardiomyotomy is a viable option as a definitive treatment for advanced megaesophagus, with relief of dysphagia in a significant number of patients, the possibility of use in more fragile patients, in addition to preventing or hindering the possible indication of esophagectomy in the future^14^.

Panchanatheeswaran et al.^26^ concluded that this surgical modality should be considered the first therapy line for patients with sigmoid megaesophagus. They also suggest that esophagectomy should be reserved for cases of cardiomyotomy failure^26^.

Costantini et al.^7^, who included in their work 1,001 patients with all-grade achalasia who underwent Heller-Dor laparoscopic surgical cardiomyotomy, concluded that there is a high probability of dysphagia relief in around 80% of these patients even 20 years after the procedure. Furthermore, they concluded that surgical complications are rare and that recurrences can be treated in most cases endoscopically, through dilation, besides obtaining acceptable rates of late reflux. On the other hand, they claim that the main predictors of unsatisfactory late results are the manometric pattern of achalasia, type III, the presence of sigmoid esophagus (2.5 odds ratio) and a high chest pain score^7^.

The recurrence of symptoms after esophageal cardiomyotomy requires thorough evaluation, as pointed out by Orlandini et al.^21^ and Tustumi et al.^37^. The rationale for classifying the condition as “persistent”, “early recurrence” and “late recurrence” is suggested, which should help guiding the diagnosis and treatment of those patients. Clinical history data and exams such as the esophagram and upper gastrointestinal endoscopy (UGE) are essential in a logical approach that can encompass diagnoses ranging from incomplete myotomy and very tight or migrated antireflux valves to neoplasia or even disease progression (megaesophagus). With reservations about the individuality of conduct in each case, after a thorough study, cases of “persistence” and “early recurrence” are more likely to require less invasive treatments, such as re-myotomy, peroral endoscopic myotomy (POEM), or even endoscopic dilation, while cases of “late recurrence” can be considered individually, for major surgeries^21,37^.

In relation to alternative and/or secondary treatments, such as POEM, Mandavdhare et al.^17^ carried out a systematic review and meta-analysis with 11 studies covering a total of 428 patients undergoing POEM for definitive treatment of advanced megaesophagus/terminal achalasia and concluded that the therapy was successful, with 89.3% clinical success after one to three years of follow-up. It is evident that POEM can be a viable alternative in cases of patients with advanced megaesophagus with recurrence of symptoms after surgical cardiomyotomy or even after re-myotomy^17^. In a recent study, Prado Junior et al.^28^ demonstrated the safety and effectiveness of the procedure, carried out systematically and by an experienced team^28^.

Regarding esophageal neoplasia – an obvious concern in patients with achalasia undergoing surgical treatment or not –, Tustumi et al.,^36^ in a meta-analysis with 11,978 patients with achalasia, concluded that there is an increased prevalence of esophageal carcinoma in this population, 28 cases for every one thousand patients. This fact is in accordance with the world literature and corroborates the need for vigilant endoscopic follow-up in patients, even after definitive surgical procedures^30^.

Finally, it should be noted that, as achalasia/advanced megaesophagus is a complex disease in itself, each case should be individualized, preferably treated by experts and in a specialized, multidisciplinary environment. The patient must be guided and informed regarding the therapeutic possibilities, expectations and risk-benefit associated with each proposed treatment modality. Due to the increased risk of neoplasia and the possibility of esophagitis, endoscopic surveillance should be performed.

The limitations of our work lie in the fact that there is a low “n” sample in the studies, which generates data inaccuracy. This is probably due to the low frequency of the disease in question. Furthermore, there is a heterogeneity of the studies discriminated. Also, different modalities of evaluation and classification of terminal achalasia/advanced megaesophagus, different periods of evaluation of late results and different modalities of evaluation of outcomes used such as questionnaires, classifications (Brandt, Eckardt), evaluation of the dysphagia symptom and levels of personal satisfaction.

Given the findings of this review study, randomized clinical trials are required to confirm them. It is not possible to determine the best profile of patients with advanced megaesophagus indicated for major surgery; however, it is estimated that they constitute a small portion of this patients’ population.

## CONCLUSIONS

This systematic review with meta-analysis allows us to conclude that patients with advanced megaesophagus can be safely treated with laparoscopic Heller cardiomyotomy with fundoplication. This surgical modality, which encompasses less complex abdominal surgery, presents high symptom resolution rates, low complication rates, low mortality rates and satisfactory results. Caveats must be considered regarding the late long-term outcome.

Even so, the present study indicates a favorable indication for the challenging surgical treatment of this complex disease. This fact can certainly guide the surgeon in his/her decision making.
